# Preventing the Development of Observationally Learnt Fears in Children by Devaluing the Model’s Negative Response

**DOI:** 10.1007/s10802-015-0004-0

**Published:** 2015-03-31

**Authors:** Gemma Reynolds, Andy P. Field, Chris Askew

**Affiliations:** 1 Department of Psychology, Kingston University, Penrhyn Road, Kingston-upon-Thames, Surrey, KT1 2EE UK; 2 School of Psychology, University of Sussex, Brighton, BN1 9RH UK; 3 Present Address: Department of Psychology, Middlesex University, The Burroughs, Hendon, London, NW4 4BT UK

**Keywords:** Childhood fear, Anxiety, Vicarious learning, Modeling

## Abstract

Vicarious learning has become an established indirect pathway to fear acquisition. It is generally accepted that associative learning processes underlie vicarious learning; however, whether this association is a form of conditioned stimulus-unconditioned stimulus (CS-US) learning or stimulus–response (CS-CR) learning remains unclear. Traditionally, these types of learning can be dissociated in a US revaluation procedure. The current study explored the effects of post-vicarious learning US revaluation on acquired fear responses. Ninety-four children (46 males and 48 females) aged 6 to 10 years first viewed either a fear vicarious learning video or a neutral vicarious learning video followed by random allocation to one of three US revaluation conditions: inflation; deflation; or control. Inflation group children were presented with still images of the adults in the video and told that the accompanying sound and image of a very fast heart rate monitor belonged to the adult. The deflation group were shown the same images but with the sound and image of a normal heart rate. The control group received no US revaluation. Results indicated that inflating how scared the models appeared to be did not result in significant increases in children’s fear beliefs, avoidance preferences, avoidance behavior or heart rate for animals above increases caused by vicarious learning. In contrast, US devaluation resulted in significant decreases in fear beliefs and avoidance preferences. Thus, the findings provide evidence that CS-US associations underpin vicarious learning and suggest that US devaluation may be a successful method for preventing children from developing fear beliefs following a traumatic vicarious learning episode with a stimulus.

Since Rachman’s (1977) original suggestion that fear of an animal, object or situation can be acquired by observing another individual’s fear of it, vicarious learning has become established as an indirect pathway to fear acquisition. Evidence of this in children comes from direct experimental investigations showing that vicarious learning can influence fear beliefs (e.g., Askew et al. 2008, 2013; Askew and Field 2007; Dunne and Askew 2013), behavioral preferences and avoidance (e.g., Askew et al. 2013; Askew and Field 2007; De Rosnay et al. 2006; Dubi et al. 2008; Dunne and Askew 2013; Egliston and Rapee 2007; Gerull and Rapee 2002), heart rate and attentional bias (e.g., Reynolds et al. 2014). However, the mechanisms underlying vicarious fear learning in humans, particularly children, are less well established.

It is generally assumed that associative learning processes underpin vicarious learning (e.g., Askew and Field 2008; Bandura 1969; Berger 1962; Davey 2002; Field 2006a; Hygge 1976; Mineka and Cook 1993; Mineka and Zinbarg 2006); however, this idea has received relatively little attention. There are essentially two possibilities for what associations are formed during vicarious learning: stimulus–response (S-R or CS-CR) learning, in which a conditioned stimulus (CS; e.g., an animal) is associated with an individual’s fear-related conditioned response (CR) to the stimulus; or stimulus-stimulus (CS-US) learning, in which a CS evokes a fear-related response via its association with an unconditioned stimulus (US). In CS-US learning, a child observing a model acting fearfully (US) in response to an animal (CS) would associate the animal with the modelled response and subsequently develop a similar fearful response to the animal. Alternatively, if CS-CR associations underpin vicarious learning, the child would associate their own fearful response with the animal.

It is often assumed that irrespective of how fears and phobias were originally acquired they can all generally be understood in terms of CS-US associations (e.g., Davey 2002; Field 2006a; Mineka and Cook 1993; Mineka and Zinbarg 2006). In both animals and humans, the contiguous presence of a US (e.g., electric shock) that evokes an unconditioned response (e.g., pain) with a neutral CS (e.g., light or buzzer) leads to the learning of associations between the CS and US, and this association mediates the CR, so that the CS comes to directly evoke a CR (e.g., fear) via a representation of the US when it is subsequently encountered alone. Similarly, in vicarious learning, an observer is assumed to associate a CS with a model’s reaction (the US) to that CS so that the CS evokes a CR related to the US. Procedurally, this assumes that vicarious learning can be understood in the same framework as direct conditioning.

Traditionally, stimulus-stimulus (CS-US) and stimulus–response learning can be dissociated using a US revaluation procedure (Rescorla 1974). US revaluation is the phenomenon whereby the severity of a US (the outcome of a learning episode) is inflated or deflated in isolation (without the presence of the CS with which it has been associated) following initial CS-US learning. This change in valuation of the US is achieved using direct exposure to, or information about, the US and results in a change in fear responses to the CS, even though the CS was never directly presented with the revalued US (Dickinson 1980; Rescorla 1974, 1980). Therefore, the strength of the CR can be dramatically modified independent of direct CS-US contingency (Davey 1989). In the animal literature, this revaluation is carried out via individual experience with the US, such as making a US more aversive by aversion learning or making it less aversive by habituating an animal to a shock US in aversive conditioning (e.g., Cleland and Davey 1982; Davey and McKenna 1983; Holland and Rescorla 1975; Rescorla 1980).

In the human literature, verbally transmitted information has been used to revalue the outcome of an initial learning event (e.g., Davey 1983, 1987, 1989; Davey and McKenna 1983). Initially participants are provided with pairings of a CS and a US until a CR is established. Next participants receive revaluation training with the US only (for example, simply being told that on future occasions, the US will be less intense). Finally, the participant is provided with test trials with the CS only. The rationale is that if a CS-US association mediates the CR, then revaluing the US will also change the CR. On the other hand, if the CR is mediated by stimulus–response (CS-CR) associations, then revaluing the US will not change the CR. Logically then, if US revaluation has an effect on a CR to a CS, it can be concluded that stimulus-stimulus associations underpin learning rather than stimulus–response associations, and therefore that the CR can be modified by experiences outside of the original learning event.

US revaluation not only provides a means of differentiating stimulus-stimulus and stimulus–response associations, but also provides a potential explanation for why intense fear may subsequently develop following even a mild learning episode. That is, later inflating the aversiveness of a US can increase the initially acquired fear-related response to an associated CS. Davey (1989) reports that acquisition of a CS-US association is independent of US revaluation, so it is possible that a CS-US association can be learnt when the US is relatively nonaversive (sensory preconditioning; Prewitt 1967; Rizley and Rescorla 1972). But if the US is later experienced alone at a greater intensity, the subsequent US inflation will result in a stronger fear response to the CS. Thus Davey (1989; 1997) has argued that US revaluation can explain one of the common criticisms of the conditioning account of fears and phobias; that not all individuals who have a phobia of a particular stimulus, object or situation, can recall a traumatic effect related to the onset of their fear (e.g., Rachman 1977). An initial traumatic experience is actually unnecessary for fear to develop later.

If vicarious fear learning is procedurally similar to CS-US learning (e.g., Mineka and Cook 1993; Mineka and Zinbarg 2006) we would expect to find US revaluation effects. A vicarious learning event would result in an observer forming an association between a representation of the CS and the model’s fearful response (US) to the CS, even if it did not directly evoke a fear response (unconditioned fear response: UR) in the observer. If the observer’s fearful response (US) is later revaluated as more aversive, the fear-related response to the associated CS would also increase (see Askew and Field 2007, 2008). That is, like in direct conditioning, US revaluation would result in the acquisition of fear after the initial, relatively mild, vicarious learning episode. Askew et al. (2008) explored the interactive effects of vicarious learning and the transmission of information by providing children with positive, negative or no verbal information about three previously unencountered animals before, during, or after a vicarious learning procedure involving the animals. Vicarious learning consisted of children seeing pictures of the animals together with pictures of faces modeling fear. Negative information provided beforehand about how threatening the animals were facilitated the effects of vicarious learning on fear beliefs. However, verbal information given either during or following vicarious learning did not affect the learning of fear beliefs. Information given following learning consisted of the fear models explaining that they were actually more scared than they appeared in the pictures (US) children had seen of them. If vicarious learning is a form of CS-US learning this inflation of the US should have increased children’s fear beliefs for animals (CS) seen with these faces. Given the results, there are two possible conclusions, either: CS-US associations are not formed during vicarious fear learning; or verbal information was an unsuccessful method for inflating the US in Askew et al.’s study.

It is important to identify the type of associations formed in vicarious learning, not just for the theoretical literature, but also for clinical interventions. Understanding learning mechanisms is crucial for establishing how phobias are acquired and also so that treatments designed to reduce children’s fear can be targeted correctly. If clinicians can identify a child’s learning history, and thus the nature of the vicariously acquired associations underlying the child’s fear, interventions can be targeted at weakening these associations.

Early research (e.g., Kravetz 1974) suggests that auditory changes in heart rate are sufficient to vicariously condition changes in listening participants’ heart rates; therefore, in the current study auditory cues were used to revalue the US. Children viewed either a fear or a neutral vicarious learning video followed by random allocation to one of three US revaluation groups. Children in the US inflation group were shown still images of the adults in the video and informed that film (with sound) of a very fast beating heart rate monitor belonged to the adult in the image. A US deflation group were shown the same images but with the sound and film of a normal heart rate. Children in the control group received no revaluation. Children’s fear beliefs, avoidance preferences and behavioral avoidance were measured following the vicarious learning and revaluation procedures. It was predicted that (a) in line with previous studies, children who experienced fear modeling but no revaluation (control group) would show increased fear beliefs, avoidance preferences, behavioral avoidance, and heart rate for the modeled animal, (b) children experiencing fear modeling followed by US inflation would show elevated fear responses compared to the fear modeling control group, and (c) children who saw fear modeling then US deflation would not show the increased responses observed in the control group. If children’s fear responses change as a result of US revaluation, then it can be argued that CS-US associations underpin vicarious learning. If there is no change, CS-CR associations are likely to underpin learning.

## Method

Children saw a vicarious learning video of either a scared or neutral model (US). Within each of these two modeling type groups, children were further assigned to one of three revaluation groups; inflation, deflation or control. Measures were taken within each child for two different animal CSs (the quokka and the cuscus), one seen in the vicarious learning video and one not, so that there was always a within child comparison between the modeled and unmodeled animal and a between groups comparison between fear modeling and neutral modeling. This design was used to ensure that there was an appropriate within-child control and resulted in a 2(modeling condition: modeled vs. unmodeled) × 2(modeling type: fear vs. neutral) × 3(revaluation: inflation, deflation, control) mixed design with modeling condition compared within children and modeling type and revaluation type compared between groups. Animals were counterbalanced across the six groups. The study was approved by the ethics committee at Kingston University.

### Participants

Ninety-four children from two schools in Essex and Suffolk, UK (46 males and 48 females), with an age range of 6 to 10 years and a mean age of 8.03 (*SD* = 0.85) years, participated in the experiment. This age range was chosen because normative fears of animals often develop around this age (e.g., Field and Davey 2001; Öst 1987). Only children with informed parental consent were able to take part in the study, and all children gave verbal assent before participating.

## Materials

With the exception of the behavioral avoidance and behavioral preferences tasks, the remainder of the experiment was automated, using a program written in E-Prime 2.0 by the first author, on a Samsung RF511 Laptop and a ProLite T2451MTS 24″ Touchscreen Monitor.

### Animal CSs

Two Australian marsupials unknown to the children, a quokka and a cuscus (see also Askew and Field 2007; Field 2006b; Field and Lawson 2003), were used in the experiment because UK children do not typically have prior experience or fear expectations for these animals (e.g., Dunne and Askew 2013).

### Fear Beliefs Questionnaire (FBQ)

Fear-related beliefs about the quokka and cuscus were measured using a computer-based Fear Beliefs Questionnaire (Field and Lawson 2003), containing seven questions for each animal; for example, “Would you be scared if you saw a quokka?” and “Would you be happy to have a cuscus for a pet?” Children responded on a 5-point Likert scale: 0 (*No*, *not at all*), 1 (*No*, *not really*), 2 (*Don*’*t know*/*Neither*), 3 (*Yes*, *probably*), and 4 (*Yes*, *definitely*). Thus there were a total of 14 questions and a mean fear beliefs score for each animal was calculated for each child. Internal consistency was high; before vicarious learning: Cronbach’s *α* = 0.86 (Quokka subscale), 0.85 (Cuscus subscale); and after vicarious learning: *α* = 0.91 and 0.88 respectively.

### Videos USs

Each child watched one silent (approx. 45 s) video clip. The video showed three female adults individually approaching a box thought to contain a quokka or a cuscus and placing their hand in the box to touch the animal. In the fear video, the females looked fearful, hesitant and nervous about placing their hand in the box. In the neutral video, the females showed no fear and happily placed their hand in the box. There were four videos in total: a fear and neutral quokka film and a fear and neutral cuscus film.

### Nature Reserve Task (NRT)

The nature reserve task (see Field and Storksen-Coulson 2007) utilized a rectangular board (size: 680 mm × 500 mm) embellished with green felt as grass, and green trees and brown fences made from pipe cleaners. Two Lego figures (a male and female) were used to represent the child.

### Touch Boxes

A touch-box behavioral avoidance task (see Askew and Field 2007; Field and Lawson 2003; Kelly et al. 2010) was used to measure avoidance of the animals and consisted of two straw-filled pet carrier boxes (size: 260 mm × 460 mm × 340 mm), with breathing holes and one large hole (diameter: 14 cm) for children to place their hand through. There was a course fabric flap on the inside of the hand hole so that children could not see into the box.

### Heart Rate Monitor

A Contec Finger Probe Pulse Oximeter was attached to the child’s finger during the touch-box task in order to measure heart rate. This portable device was selected because of its successful use in previous similar research (Reynolds et al. 2014) and its non-intrusive nature, which allows free movement to approach the boxes.

### Procedure

The setting was a school room familiar to children and each child participated individually. The whole procedure took approximately 20 min.

#### NRT 1

Children were asked to imagine that the board was a nature reserve and one of the animals (e.g., a quokka) was at one end of the reserve depicted by a photograph of the animal. Children were then asked to place a Lego figure representing them somewhere on the reserve where they would most like to be. They then placed the Lego figure on the board and the distance to the animal was measured. The same procedure was repeated for the second animal (e.g., a cuscus). The order in which the animals were presented was counterbalanced across children.

#### FBQ 1

Children sat in front of the touch-screen monitor and completed the FBQ. A picture of the animal (quokka or cuscus) was displayed in the middle of the screen to ensure children were always responding for the correct animal, and the question appeared in bold lettering across the top of the screen. Children were asked to touch one of five buttons at the bottom of the screen, ranging from 1 (*No*, *not at all*) to 4 (*Yes*, *definitely*).

#### Vicarious Learning

Children then watched either the fear or neutral video on the touch-screen monitor. Within the fear and neutral video groups, the animals were counterbalanced so that half of the children in each group saw the adult ostensibly approach a cuscus, and for the other half it was a quokka.

#### US Revaluation

Only children in the inflation and deflation groups received this phase of the study. Children in the control groups (fear video-control and neutral video-control) moved straight on to the second FBQ. All children in this phase initially saw an 8 s video clip (with audio) of a heart rate monitor displaying an accelerated heart beat (approx. 100 bpm) and were told this is what someone’s heart rate looks and sounds like when they are scared. They were then shown an 8 s video clip of a normal heart beat (approx. 68–72 bpm) and told this is what someone’s heart rate looks and sounds like when they are relaxed. Next they were told that they would see a video of the heart rate of the female models (seen during vicarious learning) when they were putting their hands in the boxes to touch the animal. Children consecutively saw one still image of each of the three females in the video paired with another 8 s video clip of a heart rate. For children in the inflation groups (fear video-inflation and neutral video-inflation), the heart rate displayed was very fast (approx. 142–150 bpm) and the sentence, ‘Wow, look how fast their heart rate is, they must have been really scared’ was displayed at the bottom of the screen. For children in the deflation groups (fear video-deflation and neutral video-deflation), children saw the normal heart rate they had earlier seen described as relaxed (approx. 68–72 bpm) and the sentence, ‘Their heart isn’t beating very fast, they couldn’t have been very scared’ was displayed at the bottom of the screen.

#### FBQ 2 and NRT 2

The children completed the FBQ and NRT a second time to determine whether fear beliefs and behavioral preferences had changed as a result of vicarious learning.

#### Behavioral Avoidance Task

Finally, children were shown two touch-boxes and told that a quokka was in one, and a cuscus was in the other. The quokka was on the left and the cuscus was on the right, and children were asked to stand on a line positioned 1 m from the boxes. They were given verbal instructions to approach the quokka and the stopwatch was started as soon as the instructions had been given. Children then had 15 s to approach the box. Heart-rate was taken at a 0 s (baseline measure), as the child approached the box (approach measure), as the child put their hand into the box (hand-in measure) and as the child withdrew their hand from the box (hand-out measure). The time it took for the child to approach the box was also recorded. If the child did not approach the box after 15 s, it was assumed that they did not wish to do so and the procedure moved on. After the first animal, children were asked to return back to the line and the same procedure was used for the second animal.

#### Debrief

Debriefing involved verbal explanations about the experiment, correct written information about the animals to ensure no false impressions had been created by the experiment, and age-appropriate worksheets to complete in order to reinforce this.

## Results

A rejection criterion of *p* < 0.05 was used for all subsequent analyses and effect sizes (*r*) are reported where interpretable and otherwise as partial eta-squared (η^2^_p_). Cohen (1988, 1992) suggestions about what constitutes a large or small effect are: *r* = 0.10 is a small effect; *r* = 0.30 is a medium effect; and *r* = 0.50 is a large effect. For partial eta-squared: η^2^_p_ = 0.02 is a small effect; η^2^_p_ = 0.13 is a medium effect; and η^2^_p_ = 0.26 is a large effect.

### Fear Beliefs

**Fig. 1:**
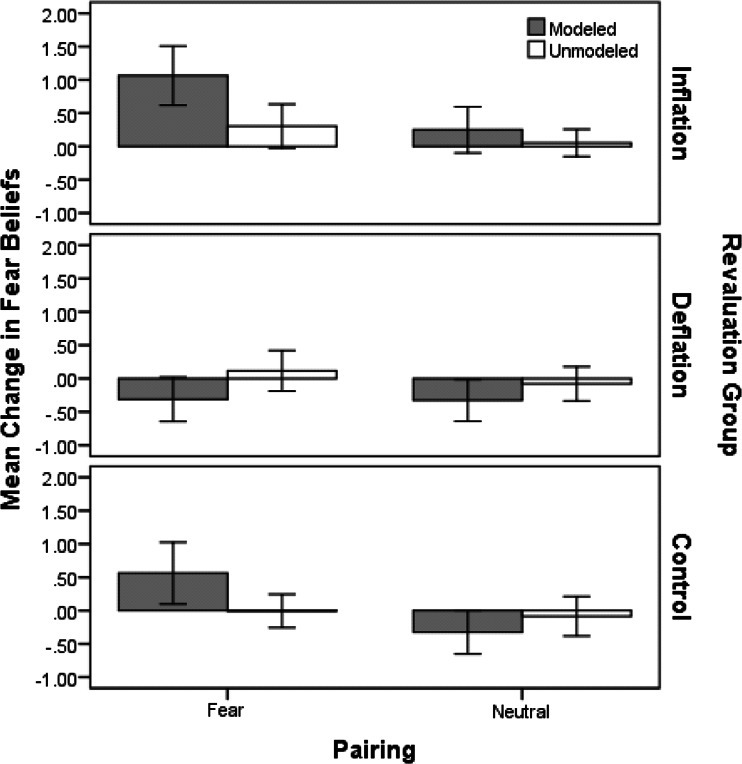
Mean (and SE) change in fear belief scores for the modeled and unmodeled animals following fear or neutral vicarious learning for each revaluation group (inflation, deflation and control)

Figure 1 displays the mean change in fear belief scores for modeled and unmodeled animals in each revaluation group following fear and neutral vicarious learning. There was an increase in children’s fear beliefs for the fear modeled animal in the inflation and control group, compared to smaller changes for unmodeled animals. Fear beliefs in the deflation group decreased for fear modeled animals and slightly increased for unmodeled animals. The graph shows that children’s fear beliefs for animals following neutral vicarious learning showed small decreases in the deflation and control groups and a slight increase in the inflation group. Changes for unmodeled animals were negligible in all three neutral revaluation groups.

A 2(modeling condition: modeled vs. unmodeled) × 2(modeling type: fear vs. neutral) × 3(revaluation: inflation, deflation, control) mixed ANOVA comparing changes in fear beliefs (FBQ2 minus FBQ1) scores indicated no significant main effect of modeling condition *F*(1, 88) = 1.49, *p* = 0.27, *r* = 0.13, but a significant main effect of modeling type, *F*(1, 88) = 12.51, *p* < 0.001, *r* = 0.35 and revaluation, *F*(2, 88) = 10.27, *p* < 0.001, η^2^_p_ = 0.19, and a significant modeling condition × revaluation interaction, *F*(2, 88) = 8.27, *p* = 0.001, η^2^_p_ = 0.16. This interaction indicates changes in fear beliefs as a result of modelling and the type of US revaluation received. It ignores whether modeling was neutral or fear-related but is interesting from a theoretical point of view because even initially neutral associations may later be revalued as more or less threatening. Thus the interaction was followed up with simple effects analyses comparing fear beliefs for modeled and unmodeled animals following inflation, deflation or no revaluation. Results indicated a significant increase in fear beliefs for the modeled animal (*M* = 0.66, 95 % CI [0.34, 0.98]) compared to the unmodeled animal (*M* = 0.18, 95 % CI [−0.02, 0.38]) following inflation, *F*(1, 91) = 10.17, *p* = 0.002, *r* = 0.32, and a significant decrease in fear beliefs for the modeled animal (*M* = −0.32, 95 % CI [−0.55, −0.09]) compared to the unmodeled animal (*M* = 0.02, 95 % CI [−0.19, 0.22]) following deflation, *F*(1, 91) = 5.09, *p* = 0.03, *r* = 0.23. However, there was no significant difference between the modeled (*M* = 0.12, 95 % CI [−0.21, 0.45]) and unmodeled animal (*M* = −0.05, 95 % CI [−0.24, 0.15]) in the control group (no revaluation), *F*(1, 91) = 1.15, *p* = 0.29, *r* = 0.11. Difference scores were created by computing the change in fear beliefs for the modeled animal minus change in fear beliefs for the unmodeled animal for each revaluation group. Follow-up *t*-tests were then carried out comparing inflation and deflation groups to the control group. Using a Bonferroni correction for carrying out two *t*-tests meant that an alpha criterion of 0.025 was used as the significance cut-off. Results indicated significant decreases in fear beliefs in the deflation group (*M* = −0.34, 95 % CI [−0.56, −0.12]) compared to the control group (*M* = 0.17, 95 % CI [−0.24, 0.58]), *t*(29) = −2.61, *p* = 0.01, *r* = 0.29, but despite evidence of a small effect, increases in the inflation group (*M* = 0.48, 95 % CI [0.20, 0.76]) compared to the control group were non-significant, *t*(29) = 1.38, *p* = 0.18, *r* = 0.21. Thus US devaluation but not inflation significantly affected children’s fear beliefs for animals that they had previously associated with a fearful or neutral model.

There was also a significant modeling condition × modeling type interaction, *F*(1, 88) = 1.86, *p* = 0.02, *r* = 0.14. This effect showed that the type of modeling (fear or neutral) given to children significantly affected their beliefs about the modeled and unmodeled animal. Finally, the modeling condition × modeling type × revaluation interaction was also significant, *F*(2, 88) = 3.15, *p* = 0.048, η^2^_p_ = 0.07, indicating that the effect of modeling condition (modeled or unmodeled) and modeling type (fear or neutral) on fear beliefs was different across revaluation groups. Further analysis was carried out to explore these differences. Simple effects analyses comparing changes in fear beliefs for modeled and unmodeled animals in each group indicated a significant change in fear beliefs for modeled animals compared to unmodeled animals in all three fear modeling groups: fear beliefs increased for the modeled animal (*M* = 1.06, 95 % CI [0.59, 1.54]) compared to the unmodeled animal (*M* = 0.30, 95 % CI [−0.05, 0.66]) in the inflation group, *F*(1, 88) = 14.07, *p* < 0.001, *r* = 0.37, and also increased for the modeled animal (*M* = 0.56, 95 % CI [0.06, 1.06]) compared to the unmodeled animal (*M* = −0.01, 95 % CI [−0.28, 0.26]) in the control group, *F*(1, 88) = 7.45, *p* = 0.008, *r* = 0.28. Fear beliefs for the modeled animal (*M* = −0.31, 95 % CI [−0.67, 0.04]) compared to the unmodeled animal (*M* = −0.12, 95 % CI [−0.21, 0.44]) decreased in the deflation group, *F*(1, 88) = 4.45, *p* = 0.04, *r* = 0.22. Following neutral modeling, there were no significant differences in the change in fear beliefs between the modeled (*M* = 0.25, 95 % CI [−0.12, −0.62]) and unmodeled animal (*M* = 0.05, 95 % CI [−0.16, 0.27]) after inflation, *F*(1, 88) = 0.94, *p* = 0.34, *r* = 0.10, deflation, *F*(1, 88) = 1.52, *p* = 0.22, *r* = 0.13 (modeled animal: *M* = −0.33, 95 % CI [−0.66, −0.004], unmodeled animal: *M* = −0.08, 95 % CI [−0.35, 0.19]), or in the control group *F*(1, 88) = 1.30, *p* = 0.26, *r* = 0.12 (modeled animal: *M* = −0.33, 95 % CI [−0.68, 0.03], unmodeled animal: *M* = −0.09, 95 % CI [−0.41, 0.23])). The results indicated then that fear beliefs increased significantly following fear modeling and fear modeling plus US inflation, and decreased significantly following fear modeling plus US deflation. Given that no significant changes were found in the neutral groups, further analyses were conducted on the fear groups only. Difference scores were created by computing the change in fear beliefs for the modeled animal minus change in fear beliefs for the unmodeled animal for each revaluation group. Follow-up t-tests were then carried out comparing inflation and deflation groups to the control group. A Bonferroni alpha criterion of 0.025 was used to correct for running two *t*-tests. Results indicated a significant decrease in the deflation group (*M* = −0.43, 95 % CI [−0.74, −0.12]) compared to the control group (*M* = 0.57, 95 % CI [−0.03, 1.18]), *t*(14) = −3.25, *p* = 0.006, *r* = 0.66, but no significant increase in the inflation group (*M* = 0.76, 95 % CI [0.31, 1.21]) compared to the control group, *t*(14) = 0.70, *p* = 0.49, *r* = 0.18.

Thus US devaluation significantly affected children’s fear beliefs irrespective of whether the original vicarious learning was fear-related or neutral: US deflation affects learnt responses even when the original CS-US association is neutral. Specific to fear-related vicarious learning, US deflation significantly lowered children’s fear beliefs compared to no revaluation; however, there was no effect of US inflation.

### Avoidance Preferences

**Fig. 2:**
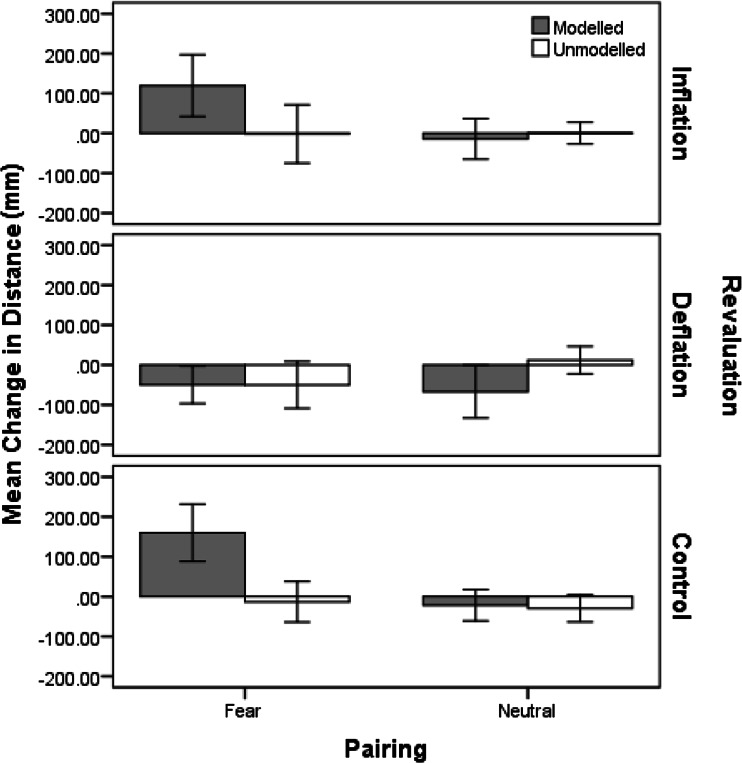
Mean (and SE) change in distance on the nature reserve task (avoidance preferences) for the modeled and unmodeled animals following fear or neutral vicarious learning for each revaluation group (inflation, deflation and control)

The maximum recorded distance from figure to animals on the nature reserve task was 680 mm; the minimum recorded distance was 0 mm, where the figure was actually touching the animal. Figure 2 demonstrates the mean change in distance (NRT 2 – NRT 1) children positioned themselves from the modeled and unmodeled animal in the nature reserve task, in each revaluation group. The graph indicates that following fear vicarious learning, children placed themselves further away from the fear modeled animal in both the inflation group and control (vicarious learning only) group, but there was practically no change for the unmodeled animal in each case. In the deflation group, children placed themselves slightly closer to both the modeled and unmodeled animals following deflation. Following neutral vicarious learning, the graph also indicates almost no change in behavioral preferences for both modeled and unmodeled animals in the inflation and control groups. In the deflation group, children placed themselves closer to the modeled animal following deflation.

A 2(modeling condition: modeled vs. unmodeled) × 2(modeling type: fear vs. neutral) × 3(revaluation: inflation, deflation, control) mixed ANOVA analysis conducted on nature reserve task change (distance post-modeling minus distance pre-modeling) scores indicated a significant main effect of modeling condition, *F*(1, 88) = 9.79, *p* = 0.002, *r* = 0.32, modeling type, *F*(1, 88) = 5.82, *p* = 0.02, *r* = 0.25, and revaluation, *F*(2, 88) = 4.75, *p* = 0.01, η^2^_p_ = 0.10. The modeling condition × revaluation interaction was also significant, *F*(2, 88) = 11.97, *p* < 0.001, η^2^_p_ = 0.21. This effect shows whether changes in avoidance preferences differ between revaluation groups for modeled animals compared to unmodeled animals but ignores whether modeling was neutral or fear-related. This effect is interesting because, theoretically, even neutral associations can subsequently be revalued. As for the fear beliefs analysis, difference scores were created by computing changes in avoidance preferences for modeled animals minus changes in avoidance preferences for unmodeled animals for each revaluation group. Follow-up t-tests comparing inflation and deflation groups to the control group indicated a significant decrease in the deflation group (*M* = −39.19, 95 % CI [−87.43, 9.06]) compared to the control group (*M* = 90.37, 95 % CI [45.68, 135.05]), *t*(29) = −4.50, *p* < 0.001, *r* = 0.37, but no significant difference in the inflation group (*M* = 53.28, 95 % CI [9.65, 96.91]) compared to the control group, *t*(29) = −1.29, *p* = 0.21, *r* = 0.21 (using a Bonferroni corrected alpha of 0.025 as the significance threshold for conducting two *t*-tests).

The modeling condition × modeling type interaction was significant, *F*(1, 88) = 32.26, *p* < 0.001, *r* = 0.56, indicating that the type of modeling (fear or neutral) significantly affected avoidance preferences for the modeled compared to unmodeled animal. Simple effects analyses showed mean increases in children’s avoidance preferences for fear modeled animals was significantly greater (*M* = 74.68, 95 % CI [28.22, 121.14]), than for (fear) unmodeled animals, which actually showed a small decrease (*M* = −21.77, 95 % CI [−57.59, 14.06]), *F*(1, 92) = 30.19, *p* = < 0.001, *r* = 0.50. Thus, indicating that, overall, fear-related vicarious learning caused increases in children’s avoidance preferences for animals. In contrast, avoidance preferences decreased for neutral modeled animal (*M* = −34.36, 95 % CI [−65.70, −3.02]) compared to the (neutral) unmodeled animal (*M* = −5.19, 95 % CI [−24.10, 13.72]) but this change was not significant, *F*(1, 92) = 2.76, *p* = 0.10, *r* = 0.17. Finally, the modeling condition × modeling type × revaluation interaction was not significant, *F*(2, 88) = 1.28, *p* = 0.29, η^2^_p_ = 0.03. Therefore the effect of fear vicarious learning compared to neutral modeling was not significantly different across revaluation groups and there was no evidence that the effect of US revaluation on avoidance preferences was any different for fear modeled animals compared to neutral modeled animals.

### Behavioral Avoidance

In total, 45 children preferred not to approach and touch the modeled animal; eight in the fear-inflation group, eight in the neutral-inflation, 11 in the fear-deflation, six in the neutral-deflation, seven in the fear-control group and five in the neutral-control group. Similarly, 42 children did not want to approach the unmodeled animal; seven in the fear-Inflation group, eight in the neutral-inflation, 10 in the fear-deflation, five in the neutral- deflation, six in the fear-control group and six in the neutral-control group. All children who did not wish to approach the animals were attributed a time of 15 s, the maximum time allowed to approach boxes. This meant that the data were non-normally distributed (negatively skewed), and this can negatively affect ANOVA calculations, particularly when sample sizes are not large.

**Fig. 3:**
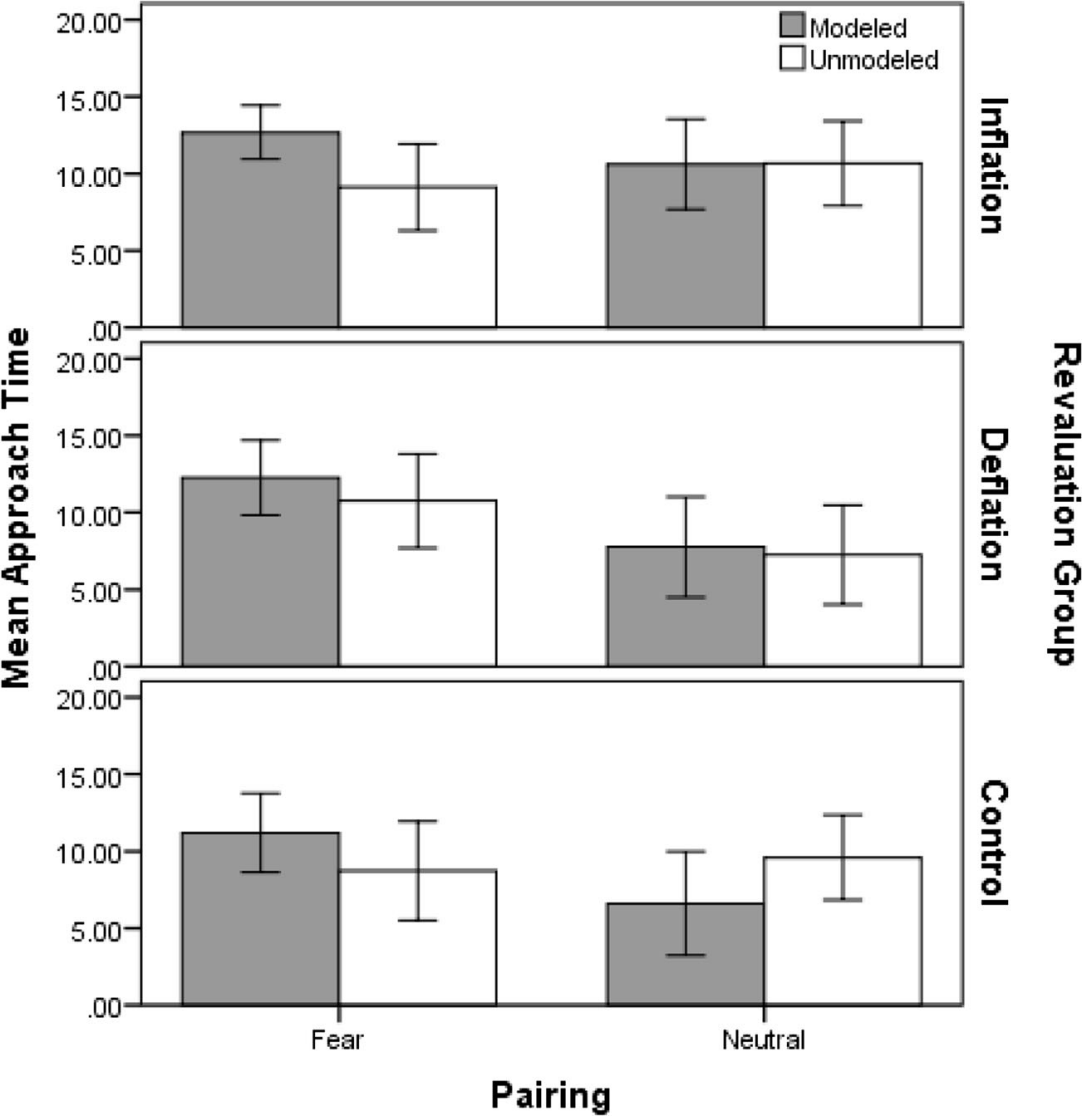
Mean (and SE) time it took to approach the modeled and unmodeled animals during the touch box task, following fear or neutral vicarious learning for each revaluation group (inflation, deflation and control)

Figure 3 shows mean approach times to modeled and unmodeled animals in each revaluation group. The graph indicates that children took slightly longer to approach the fear modeled animal compared to the unmodeled animal in all three revaluation groups. Following neutral vicarious learning though, children’s approach times were very similar for modeled and unmodeled animals in the inflation and deflation groups, but took longer to approach the unmodeled animal than the modeled animal in the control group.

A 2(modeling condition: modeled vs. unmodeled) × 2(modeling type: fear vs. neutral) × 3(revaluation: inflation, deflation, control) mixed ANOVA analysis was carried out on approach times. Results revealed that the main effect of modeling type was nonsignificant, *F*(1, 88) = 3.62, *p* = 0.06, *r* = 0.20, and there was no significant main effect of modeling condition, *F*(1, 88) = 2.93, *p* = 0.09, *r* = 0.18, or revaluation, *F*(2, 88) = 0.95, *p* = 0.39, η^2^_p_ = 0.02, or modeling condition × revaluation interaction, *F*(2, 88) = 1.46, *p* = 0.24, η^2^_p_ = 0.03. The modeling condition × modeling type interaction was significant, *F*(1, 88) = 12.11, *p* = 0.001, *r* = 0.35, indicating that the type of modeling (fear or neutral) children saw significantly affected approach times for modeled compared to unmodeled animals: approach times were longer for the fear modeled animal (*M* = 12.06, 95 % CI [10.76, 13.36]), compared to the (fear) unmodeled animal (*M* = 9.55, 95 % CI [7.81, 11.30]), and both the neutral modeled animal (*M* = 8.36, 95 % CI [6.48, 10.24]) and the (neutral) unmodeled animal (*M* = 9.17, 95 % CI [7.45, 10.89]). However, the modeling condition × modeling type × revaluation interaction was non-significant, *F*(2, 88) = 1.79, *p* = 0.17, η^2^_p_ = 0.04, indicating that behavioral avoidance was not different across revaluation groups.

### Heart Rate

Table 1 displays the mean heart rate when approaching the modeled and unmodeled animals in each revaluation group following fear and neutral vicarious learning. A 2(modeling condition: modeled vs. unmodeled) × 2(modeling type: fear vs. neutral) × 3(revaluation: inflation, deflation, control) × 4(time: baseline, approach, hand-in, hand-out) mixed ANOVA carried out on heart rate measures, revealed a significant main effect of time, *F*(3, 105) = 23.18, *p* < 0.001, η^2^_p_ = 0.40, but the main effect of modeling condition was nonsignificant, *F*(1, 35) = 3.61, *p* = 0.07, *r* = 0.31, and there was no significant modeling condition × revaluation × time interaction, *F*(6, 105) = 1.82, *p* = 0.10, η^2^_p_ = 0.09. More important though, there was a significant modeling condition × modeling type × time interaction, *F*(3, 105) = 15.79, *p* < 0.001, η^2^_p_ = 0.31, indicating that the type of modeling (fear or neutral) significantly affected heart rate over time for modeled compared to unmodeled animals. The important modeling condition × modeling type × revaluation × time interaction was also significant, *F*(6, 105) = 2.68, *p* = 0.02, η^2^_p_ = 0.13, indicating that the this effect of vicarious learning on heart rate over time differed depending on the US revaluation group children were in.

**Table 1:** Mean [and 95 % CI] heart rate when approaching the modeled and unmodeled animals during the touch box task, following fear or neutral vicarious learning for each revaluation group (inflation, deflation and control)

		Baseline	Approach	Hand-In	Hand-Out
Neutral Vicarious Learning					
Modeled Animal	Inflation	104.80 [99.39, 110.21]	104.50 [95.14, 113.86]	104.75 [95.46, 114.04]	104.75 [95.46. 114.04]
	Deflation	102.00 [93.84, 110.16]	107.11 [94.62, 119.60]	107.11 [94.62, 119.60]	106.89 [94.40. 119.38]
	Control	94.40 [85.95, 102.85]	94.80 [81.58, 108.02]	95.00 [81.75, 108.26]	95.40 [82.32, 108.48]
Unmodeled Animal	Inflation	103.80 [98.55, 109.05]	104.88 [94.87, 114.88]	105.00 [95.09, 114.91]	105.25 [94.95, 115.55]
	Deflation	101.20 [92.89, 109.51]	106.40 [95.28, 117.52]	106.60 [95.50, 117.70]	106.80 [95.45, 118.15]
	Control	94.80 [85.79, 103.81]	97.56 [84.16, 110.95]	97.78 [84.17, 111.39]	98.00 [84.42, 111.58]
Fear Vicarious Learning					
Modeled Animal	Inflation	106.71 [100.27, 113.16]	107.14 [97.60, 116.69]	108.00 [97.81, 118.19]	108.00 [97.81, 118.19]
	Deflation	102.29 [94.51, 110.06]	96.40 [84.44, 108.36]	97.20 [85.21, 109.19]	97.20 [85.21, 109.19]
	Control	102.13 [95.86, 108.41]	101.25 [91.22, 111.28]	102.75 [92.48, 113.02]	104.50 [93.75, 115.25]
Unmodeled Animal	Inflation	107.29 [100.67, 113.91]	106.50 [96.84, 116.16]	106.50 [96.84, 116.16]	106.75 [97.38, 116.12]
	Deflation	102.36 [94.11, 110.60]	99.67 [88.53, 110.81]	99.67 [88.53, 110.81]	99.67 [88.53, 110.81]
	Control	101.86 [94.79, 108.92]	100.22 [89.34, 111.10]	100.44 [89.69, 111.20]	100.67 [89.93, 111.40]

This interaction was followed up with separate 2(modeling condition: modeled vs. unmodeled) × 4(time: baseline, approach, hand-in, hand-out) mixed ANOVAs for each modeling type-revaluation group. Results for the fear modeling-inflation group indicated no significant main effect of modeling condition, *F*(1, 4) = 2.43, *p* = 0.19, *r* = 0.61, but a significant main effect of time, *F*(3, 12) = 6.85, *p* = 0.006, η^2^_p_ = 0.63, and the crucial modeling condition × time interaction, *F*(3, 12) = 4.11, *p* = 0.03, η^2^_p_ = 0.51, indicating that following fear modeling and inflation, heart rate differed over time when approaching modeled compared to unmodeled animals. However, planned comparisons comparing modeled to unmodeled animals indicated that, despite large effect sizes, children’s increase in heart rate from baseline to placing their hand in the box did not quite reach significance, *F*(1, 4) = 6.00, *p* = 0.07, *r* = 0.77, and no significant difference was found between the increase in heart rate from baseline to approaching the box, *F*(1, 4) = 4.24, *p* = 0.11, *r* = 0.72, or the increase in heart rate from baseline to withdrawing their hand from the box, *F*(1, 4) = 3.37, *p* = 0.14, *r* = 0.68 (see Table 1). Although differences in heart rate were nonsignificant, effect sizes were large and the number of children that approached both animals was very small (*N* = 5), suggesting that nonsignificance may actually have been the result of lack of power (the low *N* also suggests that these effect sizes, although large, will lack precision, making it important to replicate the effects in larger studies).

For the fear modeling-deflation group, all results were non-significant for: modeling condition, *F*(1, 4) = 0.35, *p* = 0.59, *r* = 0.28; time, *F*(3, 12) = 2.67, *p* = 0.10, η^2^_p_ = 0.40; and the modeling condition × time interaction, *F*(3, 12) = 2.67, *p* = 0.10, η^2^_p_ = 0.40. Results for the fear modeling-control group showed no significant main effect of modeling condition, *F*(1, 5) = 2.21, *p* = 0.20, *r* = 0.55, but a significant main effect of time, *F*(3, 15) = 7.09, *p* = 0.003, η^2^_p_ = 0.59, and the important modeling condition × time interaction, *F*(3, 15) = 5.66, *p* = 0.008, η^2^_p_ = 0.53, indicating that heart-rate differed across time points when approaching modeled and unmodeled animals. Planned comparisons found no significant difference from baseline to children approaching, *F*(1, 4) = 0.09, *p* = 0.77, *r* = 0.15 (see Table 1), or placing their hand in the box, *F*(1, 4) = 4.62, *p* = 0.08, *r* = 0.73. However, children’s heart rate had significantly increased from baseline to withdrawing their hand from the box, *F*(1, 4) = 8.57, *p* = 0.03, *r* = 0.83, suggesting devaluation had little effect. In order to compare any differences in effects in the three revaluation groups, a regression slope was computed for each participant over the 4 time points (baseline, approach, hand-in and hand-out) following fear vicarious learning. A mixed ANOVA 2(modeling condition: modeled vs. unmodeled) × 3(revaluation: inflation, deflation, control) was carried out on the computed betas following fear vicarious learning, revealing a significant main effect of modeling condition; *F*(1, 13) = 4.67, *p* = 0.05, *r* = 0.51, but no significant modeling condition × revaluation interaction; *F*(2, 13) = 0.07, *p* = 0.93, η^2^_p_ = 0.01. Thus the findings show the effect of modelling was not significantly different across groups.

Results for the neutral modeling-inflation group revealed a significant main effect of time, *F*(3, 21) = 3.39, *p* = 0.04, η^2^_p_ = 0.33, but no significant main effect of modeling condition, *F*(1, 7) = 0.009, *p* = 0.93, *r* = 0.04, or the critical modeling condition × time interaction, *F*(3, 21) = 1.63, *p* = 0.21, η^2^_p_ = 0.19. For the neutral modeling-deflation group, like the fear modeling-deflation group, all results were non-significant: modeling condition, *F*(1, 8) = 1.47, *p* = 0.16, *r* = 0.39, time, *F*(3, 24) = 0.31, *p* = 0.82 η^2^_p_ = 0.04, modeling condition × time interaction, *F*(3, 24) = 2.45, *p* = 0.09, η^2^_p_ = 0.24. Results for the neutral-control group were also all non-significant: animal, *F*(1, 7) = 2.03, *p* = 0.20, *r* = 0.47, time, *F*(3, 21) = 1.00, *p* = 0.41, η^2^_p_ = 0.13, animal × time interaction, *F*(3, 21) = 1.00, *p* = 0.41, η^2^_p_ = 0.13.

### Correlations

Correlation analyses were carried out to explore whether children’s baseline fear beliefs for fear modeled animals influenced subsequent learning and revaluation for the animals. For example, whether having high fear for the animals at the beginning of the experiment would be associated with greater responding to vicarious fear-learning. Baseline fear beliefs were significantly negatively correlated with changes in fear beliefs in the control group, *r*(15) = −0.54, *p* = 0.04, but not other groups. This indicates that increases in fear beliefs were larger when fear beliefs began at a lower baseline. They were also significantly correlated with pre-learning avoidance preferences in the deflation group, *r*(16) = 0.59, *p* = 0.02, and control group, *r*(15) = 0.66, *p* = 0.008. However, there were no significant correlations between baseline fear beliefs and changes in avoidance preferences. All correlations for baseline fear beliefs with behavioral avoidance and heart rate were also non-significant.

## Discussion

The current study explored the effects of US revaluation on fear responses following vicarious learning. Results for vicarious learning replicated previous research showing fear modeling leads to significant increases in fear beliefs, avoidance preferences, avoidance behavior and heart rate (e.g., Askew et al. 2008, 2013; Askew and Field 2007; Dunne and Askew 2013; Reynolds et al. 2014). One of the main aims of the study was to see if post-vicarious learning US inflation would increase scores on fear-related measures further. However, findings showed that inflating how scared the models appeared to be (i.e., the US) did not result in significantly greater increases in fear beliefs for modeled animals compared to unmodeled animals than occurred without revaluation: similar increases were found in a control group that had received the vicarious learning procedure without subsequent US revaluation. A second aim was to test whether post-learning deflation of the US would prevent increases otherwise found following fear-related vicarious learning. Deflating how scared models appeared to be not only prevented increases, but resulted in significant decreases in fear beliefs for the modeled animal compared to unmodeled animals; this decrease was significantly greater than for unrevaluated fear modelled animals, for which fear beliefs actually increased. In fact, decreases in fear beliefs for previously fear-modeled animals caused by US deflation were similar to those found for animals children had never seen with scared models. Thus US devaluation may be a successful means to prevent the development of fear beliefs following a traumatic vicarious learning episode with a stimulus.

The study also demonstrated that irrespective of whether the vicarious learning was fear-related or neutral, children showed a decrease in fear beliefs and avoidance preferences on the nature reserve task following US deflation compared to the control group. This makes sense given that children receiving neutral modeling would also, like those that had received fear modeling, form a CS-US association that may later become devalued following US deflation. Indeed no difference in devaluation was found for animals that were fear or neutral modeled. However, comparable results were not found for US inflation: despite increases in avoidance preferences following inflation for fear and neutral modeled animals, this increase was not significantly greater than for animals in the unrevaluated control group. Thus, like fear beliefs, avoidance preference results only provide support for an effect of US deflation.

Effects of US revaluation were not found for other fear measures. Following US inflation, children took longer to approach fear modeled animals compared to unmodeled animals but there was no difference across revaluation groups. Likewise, increases in children’s heart rate for fear modeled compared to unmodeled animals was only found in the control group. Therefore these results show evidence of the effect of vicarious fear learning on children’s avoidance and heart rate responses for animals, replicating Reynolds et al. (2014)) findings, but do not show evidence for a significant effect of US revaluation on these responses (though low power has contributed to this conclusion).

Only US devaluation was found to influence self-reported fear-beliefs and avoidance preferences then; no other measure showed a convincing US revaluation effect. There are several possible interpretations of these results. One interpretation is that vicarious learning is not underpinned by CS-US learning, but by S-R learning. Askew et al. (2008) also failed to find that revaluing the US with verbal information after vicarious learning affected fear beliefs for the CS. They pointed out that the US revaluation procedure they used (verbal information in the form of sound clips) may not have been potent enough to interact with the vicarious learning. The current research arguably used a more potent revaluation method by not only providing both a visual, auditory and written procedure, but also by providing the revaluation information through heart rate responses as opposed to simply telling the children the models were more, or less, scared than indicated. Research by Kravetz (1974) suggests that auditory changes in heart rate are sufficient to vicariously condition listeners’ heart rate. Moreover, the current study did not only explore the effects of US revaluation on fear beliefs, but also on additional behavioral and physiological responses. However, the fact that US devaluation effects were found for fear beliefs and avoidance preferences strongly suggests that CS-US associations underpin vicarious learning. Devaluation of the US alone, without the CS being presented again, were enough to reduce fear beliefs (CR) for the CS, suggesting that an association formed between the CS and US activated the CR. It is difficult to see how the findings could be explained by direct association between the CS and CR.

A more plausible explanation for the mixed findings might be that vicarious fear learning is only susceptible to US revaluation in certain response systems. Of Lang’s (1968) three independent fear response systems, revaluation may only influence the verbal-cognitive system (subjective report), but not the physiological or behavioral avoidance systems. The current experiment does not directly test this; however, the fact that the most convincing effects were found for fear beliefs (subjective report response system) does support this interpretation. In addition, it could be that the different effects across the different responses reflect differing sensitivities in the measures used. However, based on previous research (Reynolds et al. 2014), there is no evidence that the fear beliefs questionnaire or nature reserve task are more sensitive measures than the others used here and therefore more likely to detect US revaluation effects. Finally, it is also important to consider the possibility that the effects of US devaluation on self-reported fear beliefs and avoidance preferences are simply the result of demand characteristics. That is, children may have deliberately modified their responses to reflect what they believed researchers wanted them to do. Clearly, this is more likely to occur in a self-report measure than a measure of heart rate responses, which are arguably very difficult to consciously control. However, this does not convincingly explain the lack of US inflation effects, which we might also expect to show parallel changes because of demand features. That all learning effects were the result of demand characteristics is also extremely unlikely given that general vicarious learning effects were also found for heart rate, which is arguably very difficult to consciously modify.

The current findings are important because they indicate that fear-related beliefs and avoidance preferences following vicarious learning may be modified by experiences after the original learning event. In particular, US deflation decreased children’s fear-beliefs for animals immediately following a vicarious fear learning episode. In contrast, the fear beliefs of children who did not have US devaluation increased, indicating that early US devaluation is likely to be an effective means of reducing or preventing the development of vicariously acquired fear cognitions (Davey 1989). For example, if a parent or teacher recognizes that a child has witnessed a peer responding fearfully (US) to a stimulus (CS) they could seek to devalue that peer’s response and prevent fear developing. Similarly the findings suggest that if a parent or teacher has themselves shown fear of something in front of a child, devaluing their fear response is likely to be beneficial to the child. One of the strengths of the current paradigm is that it enables investigation of revaluation directly after the learning experience. The findings are therefore specific to early intervention and the prevention of fear. From a clinical perspective, it would also be important to explore whether deflating the US at a later time point would reduce established fear (i.e., fear treatment rather than prevention).

More generally, the fact that CS-US associations underpin vicarious learning suggests ways that prevention and treatment interventions might be developed to specifically target these associations. Much is already known about the properties of CS-US associations; for example, the contingency between a CS and US is known to be critical for associative learning (see e.g., Field 2006a). Prior positive learning history with a stimulus is likely to prevent future vicarious fear learning by weakening the contingency between the stimulus and any fear-related outcome (US). Thus educational or experiential interventions that develop a positive learning history for a stimulus are likely to prevent later fear development. Similarly, when vicarious learning has already occurred, treatment could be focused on weakening learnt CS-US associations. US devaluation and commonly used techniques such as extinction and counterconditioning are likely to be efficacious, but factors such as learning context and CS salience may also play an important role.

From a clinical perspective, understanding learning mechanisms is potentially critical for designing more effective interventions for reducing children’s fear (Field 2006a). In practice though, the acquisition of individual fears and phobias are likely to be determined by a complex set of factors and not merely be the result of one causal influence such as vicarious learning. Fear development might involve both direct and indirect learning along with other factors including genetic, biological, and parenting influences (e.g., Muris and Merckelbach 2001). Therefore, any interpretation of the results needs to be considered in the context of this wider set of influences. Additionally, given that a non-clinical sample of children was investigated, only cautious generalizations to clinically anxious populations can be made. On the other hand, one of the strengths of the vicarious learning paradigm is that it represents a non-harmful analogue of real world learning that can be used with non-fearful children.

To summarize, the findings indicate that US devaluation can reduce fear cognitions for animals following fear-related vicarious learning, implying that CS-US associations underpin vicarious learning in children. Thus the research adds crucial information to our current understanding of the underlying mechanisms of vicarious learning and has important implications for interventions to prevent fear development.
